# Factors associated with hospitalization in the acute phase of Chikungunya

**DOI:** 10.1371/journal.pone.0296131

**Published:** 2023-12-22

**Authors:** Danielle Torres dos Santos Lopes, Crispim Cerutti Junior, Aline Areias Cabidelle, Angelica Espinosa Miranda, Iuri Drumond Louro, Luciano Pamplona de Góes Cavalcanti, Creuza Rachel Vicente

**Affiliations:** 1 Postgraduate Program in Infectious Disease, Federal University of Espírito Santo, Vitória, Espírito Santo, Brazil; 2 Department of Social Medicine, Federal University of Espírito Santo, Vitória, Espírito Santo, Brazil; 3 Health Surveillance Sector, Health Department of Vitória, Vitória, Espírito Santo, Brazil; 4 Postgraduate Program in Biotechnology, Federal University of Espírito Santo, Vitória, Espírito Santo, Brazil; 5 Department of Biology, Federal University of Espírito Santo, Vitória, Espírito Santo, Brazil; 6 Postgraduate Program in Collective Health, Federal University of Ceará, Fortaleza, Ceará, Brazil; University of South Florida, UNITED STATES

## Abstract

**Objective:**

Determine characteristics associated with hospitalization in the acute phase of Chikungunya.

**Methods:**

Cross-sectional study including data on Chikungunya cases reported in Vitória, Espírito Santo state, Brazil, between March 2016 and December 2021.

**Results:**

Hospitalizations accounted for 1.42% (n = 41) of the 2,868 cases included. There were statistically significant differences between hospitalized and non-hospitalized regarding age (*P* 0.001), which was lower among hospitalized patients, and pregnancy, which was more frequent in the hospitalized group (*P* 0.010). Patients younger than two years old and older than 65 years corresponded to 31.7% of hospitalizations. Back pain (OR = 0.134; 95% CI = 0.044–0.409) and arthralgia (OR = 0.226; 95% CI = 0.083–0.613) were protective factors for hospitalization.

**Conclusion:**

Groups at risk of severe Chikungunya, including those under two and over 65 years of age, may require more hospitalization, even with milder manifestations.

## Introduction

Chikungunya is a neglected tropical disease caused by an RNA virus of the family Togaviridae, genus Alphavirus, which is transmitted to humans through the bite of infected mosquitoes of the species *Aedes aegypti* and *Aedes albopictus* [1]. Authoctonous transmission has been reported in 114 countries and independent territories where 77.9% of the world population lives, with an annual estimate of global clinical cases varying from 52,774 to 328,943 between 2010 and 2019 [2], having a rapid expansion in recent years [3].

The signs and symptoms more frequent in the acute phase of Chikungunya are high fever lasting three to five days, polyarthralgia, polyarthritis, edema in hands and feet, moderate to intense myalgia, headache, and rash [4]. Decompensation of comorbidities has been frequently observed in the acute phase [5]. In addition, atypical manifestations may affect patients, such as neurological, cardiological, hepatic, renal, pulmonary, and hematological presentations [5]. These manifestations may be related to the direct effect of the virus, the immunological response, or the toxicity of the medication [6]. Severe forms may affect different age groups [7], and hospitalizations during outbreaks have been reported to vary from 0.5% to 8.7% of the cases [8, 9]. Nevertheless, a change in the severity profile, with increasing deaths, has been observed in recent outbreaks [10].

The increasing incidence, spatial distribution, and the number of severe and atypical cases of Chikungunya reinforce the importance of understanding the factors related to hospitalization, contributing to clinical management and public health policies. Therefore, the objective of the present study was to determine the demographical and clinical characteristics associated with hospitalization in the acute phase of Chikungunya.

## Material and methods

### Study design

This cross-sectional study included data on Chikungunya cases reported in Vitória, the capital of Espírito Santo state, located in the Southeast region of Brazil, between March 2016 and December 2021. For cases reported up to March 31, 2020, data was obtained through the Notifiable Diseases Information System (SINAN) and those reported after April 01, 2020, through the eSUS Health Surveillance (eSUS-VS).

### Inclusion and exclusion criteria

Cases included in the study were residents of Vitória, reported in the acute phase of Chikungunya, and presented laboratory confirmation of the infection. The acute clinical presentation met the following criteria: sudden-onset fever and intense polyarthralgia, usually accompanied by back pain, skin rash, headache, and fatigue, with an average duration of seven days [6]. Cases with laboratory confirmation presented at least one of the following criteria: positive viral isolation; detection of viral RNA by RT-PCR; detection of IgM in a single serum sample collected during the acute or convalescent phase; demonstration of seroconversion—negative to positive or fourfold increase in IgG titers by serological tests (ELISA or hemagglutination inhibition test) between samples in the acute (first eight days of illness) and convalescent (preferably 15 to 45 days) phases after the onset of symptoms, or 10 to 14 days after sample collection in the acute phase) [5].

Exclusion criteria comprised cases without data about hospitalization and duplicates. Duplicates were determined by checking the date of birth and the mother’s name, and only the first report remained in the databank.

### Definition of variables

The following variables were evaluated: age; sex; pregnancy; days of disease evolution (from the onset of signs and symptoms to the report); clinical manifestations (fever, myalgia, headache, rash, vomiting, nausea, back pain, conjunctivitis, arthritis, arthralgia, petechiae, leukopenia, positive tourniquet test, retroorbital pain); pre-existing diseases (diabetes, hematological diseases, liver diseases, chronic kidney disease, arterial hypertension, acid-peptic disease, autoimmune diseases); hospitalization; days until hospitalization (from the onset of signs and symptoms to hospitalization).

Age was measured in years and categorized according to recognized risk groups (newborn, younger or equal to two years old, and older or equal to 65 years old) and decades.

Pre-existing diseases were defined by ongoing treatment or specific diagnosis and were mentioned by the clinician in the Chikungunya report form.

### Statistical analysis

Absolute and relative frequencies represented the categorical variables, and the Pearson Chi-Square test or Fisher Exact test were used to compare these variables regarding hospitalization status. Medians and interquartile ranges represented the continuous variables, and the Mann-Whitney test was used to compare them regarding hospitalization status. A *P* lower than 0.005 was considered significant.

The multivariate binary logistic regression results using the Forward Stepwise method were presented as *Odds Ratio* (OR) with a 95% confidence interval (95% CI). Data were analyzed using the software SPSS® version 20 (IBM®, New York, USA).

### Ethical statement

The Research Ethics Committee of the Health Center Sciences at the Federal University of Espírito Santo approved the study protocol (opinion number 4.393.656). The procedures followed the ethical standards of the responsible committee on human experimentation and the Helsinki Declaration of 1975, as revised in 2000. The Ethics Committee waived the need for consent. The data were accessed on 17 September 2021, and the authors had access to information identifying individual participants during data collection.

## Results

**Fig 1:**
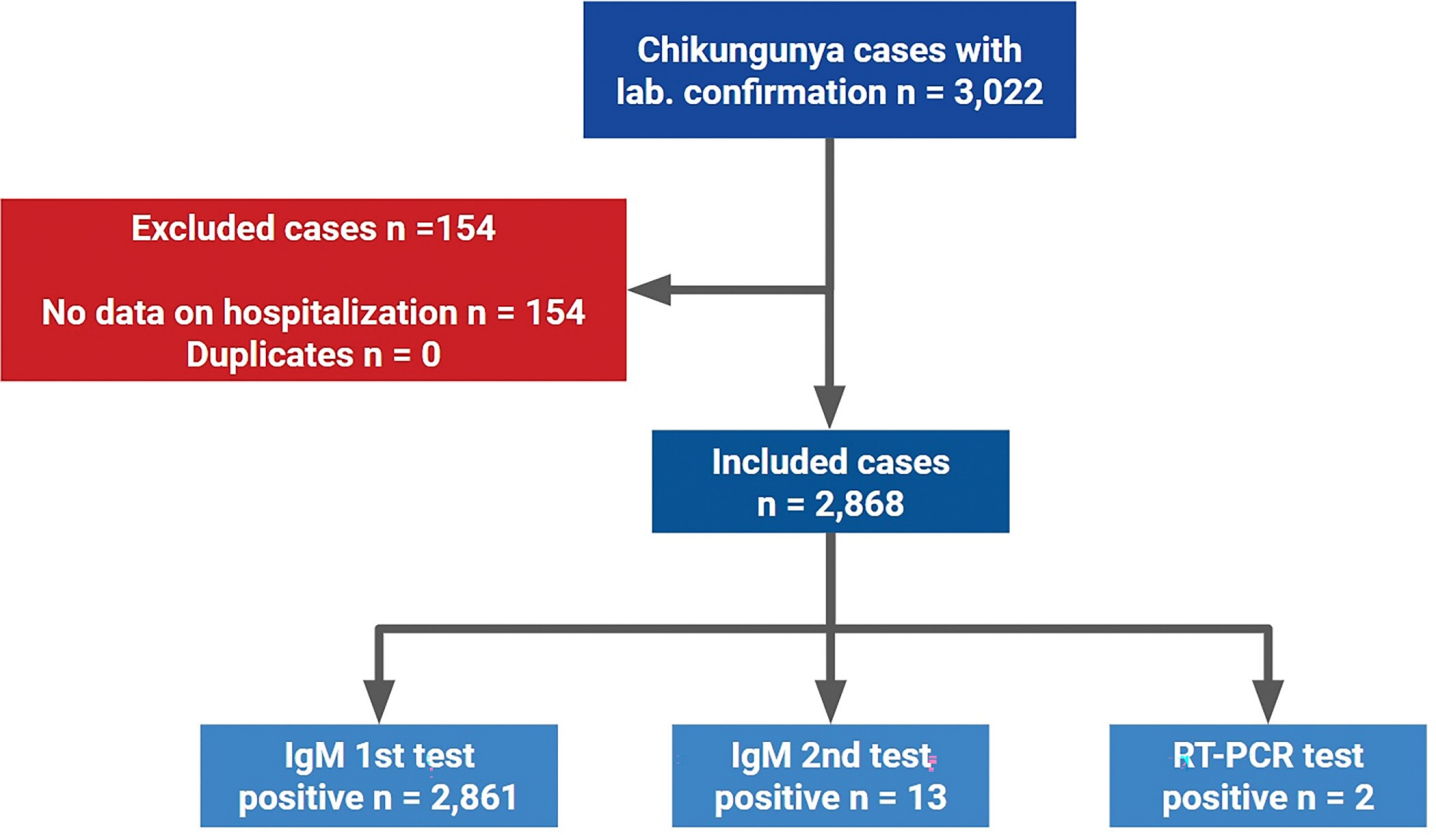
Composition of the study sample. Eight patients tested positive in more than one test.

Between 2016 and 2020, Vitória municipality had 3,022 reports of acute cases of Chikungunya with laboratory confirmation. Of these, 154 were excluded due to the absence of data about hospitalization. No duplicate was identified. Of the 2,868 cases included, 2,861 were positive in the IgM test in the first sample collection and thirteen in the second. Two cases were positive in RT-PCR. Eight patients tested positive in more than one test (Fig 1).

Most of the cases occurred in women (67.5%), with a median age of 47 years (interquartile range = 34–59), presenting as main symptoms arthralgia (91.6%), myalgia (89.0%), fever (87.9%), headache (80.4%) and back pain (65.2%). There was no missing data for variables related to demographic characteristics and signs and symptoms. The most frequently pre-existing diseases were arterial hypertension (28.4%) and diabetes (13.4%). Nevertheless, six patients had no data about liver diseases, five for diabetes, chronic kidney disease, hypertension, acid peptic disease, autoimmune disease, and four for hematological diseases (Tables 1 and 2).

**Table 1:** Demographic characteristics of Chikungunya cases according to hospitalization status.

	Hospitalized	Non-hospitalized	OR (95% CI)*	Total
	N = 41	N = 2,827		N = 2,868
**Sex**	n (%)	n (%)		n (%)
Female	26 (63.4)	1,910 (67.56)	0.832 (0.439–1.579)	1,936 (67.5)
Male	15 (36.6)	917 (32.44)		932 (32.5)
**Age**				
Median (IQR)	26 (12–61)	47 (34–59)	0.001**	47 (34–59)
Newborn	3 (7.3)	5 (0.2)	**-**	8 (0.3)
≤ 2 years	2 (4.9)	5 (0.2)	**-**	7 (0.2)
3 to 9 years	4 (9.8)	59 (2.1)	**-**	63 (2.2)
10 to 19 years	8 (19.5)	179 (6.3)	**-**	187 (6.5)
20 to 29 years	5 (12.2)	267 (9.4)	**-**	272 (9.5)
30 to 39 years	4 (9.8)	470 (16.6)	**-**	474 (16.5)
40 to 49 years	1 (2.4)	536 (19.0)	**-**	537 (18.7)
50 to 59 years	3 (7.3)	606 (21.4)	**-**	609 (21.2)
60 to 64 years	3 (7.3)	273 (9.7)	**-**	276 (9.6)
≥ 65 years	8 (19.5)	427 (15.1)	**-**	435 (15.2)

IQR = Interquartile range

*Crude *Odds Ratio* with 95% Confidence Interval

**Mann-Whitney U test.

**Table 2:** Clinical characteristics of Chikungunya cases according to hospitalization status.

	Hospitalized	Non-hospitalized	OR (95% CI)*	Total
	N = 41	N = 2,827		N = 2,868
**Clinical manifestations**	n (%)	n (%)		n (%)
Fever	34 (82.9)	2,488 (88.0)	0.662 (0.291–1.505)	2,522 (87.9)
Myalgia	31 (75.6)	2,521 (89.2)	**0.376 (0.183**–**0.775)**	2,552 (89.0)
Headache	19 (46.3)	2,286 (80.9)	**0.204 (0.110**–**0.380)**	2,305 (80.4)
Rash	9 (22.0)	949 (36.6)	0.557 (0.265–1.171)	958 (33.4)
Vomiting	11 (26.8)	484 (17.1)	1.175 (0.883–3.566)	495 (17.3)
Nausea	11(26.8)	1,335 (47.2)	**0.410 (0.205**–**0.821)**	1,346 (46.9)
Back pain	9 (22.0)	1,862 (65.9)	**0.146 (0.069**–**0.307)**	1,871 (65.2)
Conjunctivitis	0 (0.0)	310 (11.0)	-	310 (10.8)
Arthritis	9 (22.0)	1,155 (40.9)	**0.407 (0.194**–**0.856)**	1,164 (40.6)
Arthralgia	31 (75.6)	2,595 (91.8)	**0.277 (0.134**–**0.572)**	2,626 (91.6)
Petechiae	5 (12.2)	346 (12.2)	0.996 (0.388–2.555)	351 (12.2)
Leukopenia	7 (17.1)	412 (14.6)	1.207 (0.531–2.741)	419 (14.6)
Positive tourniquet test	1 (2.4)	336 (11.9)	0.185 (0.025–1.353)	337 (11.8)
Retroorbital pain	4 (9.8)	1,268 (44.9)	**0.133(0.047**–**0.374)**	1,272 (44.4)
**Pre-existing diseases** ^#^				
Diabetes	7/41 (17.1)	378/2,822 (13.4)	1.331 (0.586–3.024)	385/2,863 (13.4)
Hematological diseases	0/41 (0.0)	13/2,823 (0.5)	-	13/2,864 (0.5)
Liver diseases	1/41 (2.4)	27/2,821 (1.0)	2.587 (0.343–19.506)	28/2,862 (1.0)
Chronic kidney disease	1/41 (2.4)	14/2,822 (0.5)	5.014 (0.644–39.053)	15/2,863 (0.5)
Arterial hypertension	10/41 (24.4)	805/2,822 (28.5)	0.808 (0.394–1.656)	815/2,863 (28.5)
Acid-peptic disease	1/41 (2.4)	32/2,822 (1.1)	2.180 (0.291–16.344)	33/2,863 (1.2)
Autoimmune diseases	0/41 (0.0)	51/2,822 (1.8)	-	51/2,863 (1.8)

*Crude *Odds Ratio* with 95% Confidence Interval

^**#**^Six patients had no data about liver diseases, five for diabetes, chronic kidney disease, hypertension, acid peptic disease, autoimmune disease, and four for hematological diseases. The numbers of available data on pre-existing diseases for each group are described together with the positive cases.

Hospitalizations accounted for 1.42% of the sample, with 41 cases, mostly in females (63.4%), with a median age of 26 years (interquartile range = 12–61). The most frequent signs and symptoms in hospitalized patients were fever (82.9%), myalgia (75.6%), and arthralgia (75.6%), and the most common pre-existing diseases were arterial hypertension (24.4%) and diabetes (17.1%). The median days of disease evolution were five (interquartile range = 2–13), and the median days until hospitalization were two (interquartile range = 1–6) (Tables 1 and 2).

The reasons for hospitalization were thrombocytopenia (n = 6; 14.63%), diarrhea (n = 4; 9.75%), and syncope (n = 2; 4.87%). In 28 (68.29%) patients, it was not possible to identify the reason for hospitalization, although comorbidities such as asthma (n = 2; 4.87%) and heart disease (n = 1; 2.43%) were reported. In addition, three hospitalizations occurred in newborns (7.3%), two in children under two years old (4.9%), and eight in people over 65 years old (19.5%). Twenty-four hospitalizations occurred in public hospitals (n = 24; 58.53%), nine in private hospitals (21.94%), and eight did not present this information (19.51%).

Of the 2,827 non-hospitalized cases, the majority were female (67.6%), with a median age of 47 years (interquartile range = 34–59). The main symptoms in them were arthralgia (91.8%), retro-orbital pain (91.8%), myalgia (89.2%), fever (88.0%), headache (80.9%), back pain (65.9%), nausea (47.2%), arthritis (40.9%) and the most frequent pre-existing diseases were arterial hypertension (28.5%) and diabetes (13.4%). The median days of disease evolution in non-hospitalized patients were four (interquartile range = 1–11) (Tables 1 and 2).

Regarding pregnancy, among the 26 females hospitalized, 19 were of childbearing age, and of those, two were pregnant (10.5%). Among the 1,910 non-hospitalized females, 1,792 were of childbearing age, with 36 pregnancies (2.0%).

There were statistically significant differences between hospitalized and non-hospitalized regarding age (*P* 0.001), which was lower among hospitalized patients, and pregnancy, which was more frequent in the hospitalized group (*P* 0.010, OR = 5.739, 95% CI = 1.278–25.766). In addition, some clinical manifestations were significantly less frequent among hospitalized patients, such as myalgia (*P* 0.006), headache (*P* 0.000), nausea (*P* 0.009), back pain (*P* 0.000), arthritis (*P* 0.014), arthralgia (*P* 0.000) and retro-orbital pain (*P* 0.001). There was no statistically significant difference between the groups regarding sex (*P* 0.573), pre-existing diseases, and days of disease evolution (*P* 0.102) (Tables 1 and 2).

Multivariate logistic regression was performed to determine whether age, pregnancy, myalgia, headache, nausea, back pain, arthritis, arthralgia, and retro-orbital pain were predictors for hospitalization. The model that included back pain and arthralgia was found to be significant [X2(2) = 25.378; P < 0.000, R2 Negelkerke = 0.127]. Notably, both back pain (OR = 0.134; 95% CI = 0.044–10.409) and arthralgia (OR = 0.226; 95% CI = 0.083–0.613) emerged as significant predictors (Table 3).

**Table 3:** Final model with factors associated with hospitalization in the acute phase of Chikungunya.

Variable	OR*	95% Confidence Interval	
		Lower limit	Upper limit
Back pain	0.134	0.044	0.409
Arthralgia	0.226	0.083	0.613

Multivariate binary logistic regression using the Forward Stepwise method. Variables not included in the model: Age, pregnancy, myalgia, headache, nausea, arthritis, and retro-orbital pain. Negelkerke = 0.127

*Adjusted *Odds Ratio* with 95% Confidence Interval.

## Discussion

The study demonstrated that hospitalization is uncommon in the acute phase of Chikungunya infection, as described previously [8, 9]. Age, some clinical manifestations, and pregnancy significantly differed between hospitalized and non-hospitalized patients. However, only some clinical manifestations remained predictors of hospitalization, demonstrating milder signs and symptoms in hospitalization cases than in non-hospitalizations, corroborating a previous study [8]. Since the time to seek treatment did not differ between the groups, with a variation of four to five days, as found in previous investigations [11, 12], this factor probably did not contribute to the outcome.

Back pain and arthralgia, which were protective predictors of hospitalization, and other symptoms that were significantly less common in hospitalized patients, such as headache, nausea, back pain, arthritis, and retro-orbital pain, are self-reported by patients. Therefore, individual aspects interfering with this report, such as young age, may have influenced the results. In the present study, 19.5% of hospitalized patients in Vitória had less than two years old, being three newborns, which partially explained the milder manifestations in hospitalized patients and contributed to the significantly younger age in hospitalized group compared with non-hospitalized. Young children present a higher risk of developing severe Chikungunya [5, 8, 13]. Newborns with the infection may have neurological complications, hemorrhage, cardiac involvement, and sequels affecting cognitive development [5]. Diarrhea, which may occur in children but is rare in adults, was a reason for hospitalization in the present study [1]. In addition, thrombocytopenia was described in hospitalized children with Chikungunya infections and cited in Vitória as a reason for hospitalization [14, 15]. Some symptoms more typical in children must be considered in Chikungunya diagnosis, such as high prolonged fever, irritability, and vesicular-bullous rash [14–16].

Pregnant women had a higher risk of hospitalization and are a group under attention also due to mother-to-child transmission, especially in the intrapartum period [9, 17]. Higher risk of hospitalization [18], reports of abortions [19], neonatal deaths [17], urgent cesareans due to fetal distress [20], and sepsis with hypoperfusion and organ dysfunction [19] were previously reported in Chikungunya virus infection during pregnancy. Nevertheless, discordant results show no effects of the infection on pregnancy outcomes [9, 18], indicating that more investigations are necessary in this group.

Among hospitalized patients, 19.5% were over 65 years old, also a population at higher risk of severe Chikungunya [5, 8] due to previous diseases and low natural immune response [2, 6, 21–23]. Signs of severity are conditions that define clinical conduct and criteria for hospitalization [5]. Severity may be related to hemodynamic failure, pain not controlled by tramadol and codeine, bleeding, and comorbidities that can decompensate or lead to atypical presentations [24].

Asthma and heart disease were conditions described in hospitalized patients that also contribute to Chikungunya severity [13, 25]. Although, no differences were found in hospitalized and non-hospitalized groups regarding pre-existing diseases, possibly due to the small sample of hospitalized patients. Arterial hypertension and diabetes were the most prevalent pre-existing diseases in both groups, corroborating previous studies [22, 25, 26], that also found an association between them and disease severity [6, 21, 27], clinical instability [28], increased systemic complications [28], and hospitalization [2, 25, 29]. In addition, a previous study indicated that chronic kidney disease also increases hospitalization for Chikungunya infection [30]. Manifestations unrelated to Chikungunya may also be reasons for hospitalization during the disease and must be considered [8].

The population of the present study was very similar to previous ones, with a predominance of young adults [8, 25, 31] and females [25, 31, 32], reflecting the local demography and the use of health services. Most of the cases occurred during the COVID-19 pandemic, which may have affected the seeking of treatment behavior, the quality of the reports and imposed additional challenges to the diagnosis [33]. Therefore, the exclusive inclusion of laboratory-confirmed cases in the present study was conservative to avoid selection bias.

The study limitations are inherent to the use of secondary data, such as missing or imprecise data, especially regarding the reasons for hospitalizations and pre-existing diseases. In addition, the small sample of hospitalized patients affects the study power. Considering that hospitalization is uncommon for Chikungunya infection, future investigations should be benefited from a multicentric approach.

Due to the increasing incidence of Chikungunya, hospitalizations are expected to rise. Groups at risk of severe Chikungunya, including those under two and over 65 years of age, may require more hospitalization, even with milder manifestations during the acute phase of the disease, requiring the attention of health professionals and preparation of health services. Additional investigations are necessary to reach a definite conclusion about these findings due to the limited sample of hospitalization in the present study. Nevertheless, the results highlight potential factors contributing to better clinical management and health resource planning, particularly in Chikungunya outbreaks.
